# Associations Between Dissociation and Childhood Trauma in Borderline Personality Disorder and Affective Disorders: A Network Analytic Approach

**DOI:** 10.1111/acps.70131

**Published:** 2026-08-09

**Authors:** Philipp Wülfing, Carsten Spitzer, Judy Leibowitz, Stephen Pilling, Peter Fonagy, P. Read Montague, Tobias Nolte

**Affiliations:** ^1^ Department of Psychosomatic Medicine and Psychotherapy University Medical Center Rostock Rostock Germany; ^2^ Research Department for Clinical, Educational and Health Psychology University College London London UK; ^3^ Fralin Biomedical Research Institute at VTC, Virginia Tech Roanoke Virginia USA; ^4^ Anna Freud London UK

**Keywords:** affective disorders, borderline personality disorder, childhood trauma, dissociation, network analysis, posttraumatic stress disorder

## Abstract

**Background:**

Dissociation and childhood trauma are strongly associated with borderline personality disorder (BPD), yet it remains unclear whether this reflects disorder‐specific mechanisms or differences in symptom severity within a shared transdiagnostic structure.

**Objective:**

Using network analysis, we examined whether the conditional dependency architecture linking childhood trauma, dissociation, posttraumatic stress symptoms, and general psychological distress differs between individuals diagnosed with BPD and affective disorders (AD).

**Method:**

Participants were drawn from a large outpatient clinical sample and matched on age, gender, and global symptom severity (BSI‐GSI). Networks were estimated using regularized partial correlation models including CTQ subscales, DES subscales, PTSD symptom clusters, and overall distress. Network Comparison Tests assessed structural invariance and global strength differences between diagnostic groups.

**Results:**

As expected, individuals with BPD reported significantly higher levels of childhood trauma and dissociation than those with AD. However, despite these marked differences in severity, the overall network structure did not significantly differ between groups, and global connectivity was comparable. Bridge centrality analyses indicated similar patterns of interconnection across trauma, dissociation, and PTSD domains in both diagnoses.

**Conclusions:**

These findings suggest that BPD is characterized by elevated severity within a largely shared trauma–dissociation architecture, rather than by a qualitatively distinct network configuration. The results support a transdiagnostic model in which childhood trauma and dissociative processes are embedded within a common psychopathological structure, with diagnostic distinctions reflecting differences in magnitude rather than rewiring of interrelations. This severity–versus–architecture distinction has implications for understanding mechanisms of vulnerability and for targeting shared pathways across diagnostic categories.

## Highlights

- Significant outcomes.
  ○ Individuals with BPD reported substantially higher levels of childhood trauma and dissociative symptoms than individuals with affective disorders; however, the conditional network structure linking trauma, dissociation, PTSD symptoms, and psychological distress did not differ significantly between groups.
  ○ The findings support a severity‐within‐architecture account, suggesting that BPD may reflect greater symptom burden within a shared trauma‐related vulnerability system rather than a qualitatively distinct trauma–dissociation network organization.
  ○ The largely comparable network architecture across diagnoses supports transdiagnostic approaches targeting shared trauma‐related mechanisms, including dissociation, trauma processing, and emotion regulation, while acknowledging differences in clinical severity and complexity.
- Limitations.
  ○ The cross‐sectional design precludes causal inferences regarding the temporal relationships between childhood trauma, dissociation, and psychopathology.
  ○ Diagnostic heterogeneity may have influenced the findings, as affective disorders were not systematically assessed in the BPD sample, personality disorders were not formally excluded in the AD sample, and diagnoses were based on routine clinical practice rather than standardized research interviews.
  ○ Childhood trauma was assessed retrospectively using self‐report measures and may therefore be subject to recall bias.

## 1 Background

Childhood trauma is a well‐established risk factor for dissociative symptoms [1], and both constructs have been implicated in the etiology and clinical expression of multiple mental disorders, including borderline personality disorder (BPD) and affective disorders (AD). Dissociation refers to a “disruption of and/or discontinuity in the normal integration of consciousness, memory, identity, emotion, perception, body representation, motor control, and behavior” ([2], 291). While trauma‐related models are well supported [1], contemporary perspectives emphasize that dissociation arises from a multifactorial interplay of trauma exposure, individual vulnerability, and cognitive‐contextual mechanisms [3, 4]. Consistent with this view, dissociation occurs across a wide range of psychiatric conditions and is associated with functional impairment and clinical burden [5, 6].

Childhood trauma represents a transdiagnostic risk factor for adult psychopathology, predicting increased rates of affective, anxiety, bipolar, and psychotic disorders [7], with cumulative exposure showing dose–response relationships with psychiatric outcomes and functional impairment [8]. Childhood trauma is also associated with dissociative symptoms across diagnostic categories, supporting the conceptualization of dissociation as a trauma‐related response pattern [9]. Within this framework, BPD represents a relevant condition, as dissociative symptoms are highly prevalent, with many individuals exhibiting clinically relevant dissociation or comorbid dissociative disorders [10, 11]. Meta‐analytic evidence further indicates elevated rates of childhood trauma in BPD compared to nonclinical controls (OR ≈ 14), particularly for emotional abuse and neglect [12]. These findings support developmental and attachment‐based models of BPD that emphasize the role of early interpersonal trauma in shaping affect regulation, self‐concept, and interpersonal functioning [3, 13] and position BPD as a paradigmatic condition for studying trauma‐dissociation links in adulthood. Empirical work has consistently linked greater dissociative experiences in BPD to more severe childhood trauma, with evidence suggesting that the strength of these associations varies across trauma subtypes and exposure characteristics [9, 14]. In this context, meta‐analytic evidence indicates that individuals with BPD report childhood trauma more frequently than both nonclinical and psychiatric control groups, with particularly strong associations observed for emotional abuse and emotional neglect [12].

Stronger associations have been reported for sexual and physical abuse and for earlier or prolonged exposure, whereas emotional neglect may be particularly relevant [12]. Dissociation in BPD has been conceptualized within structural dissociation frameworks as reflecting disruptions in the integration of self‐related experiences and personality functions [3, 13, 15] and is closely linked to core features of the disorder, including affective instability and identity disturbance [16, 17, 18]. It is also associated with alterations in emotional and cognitive functioning, including attention, memory, and emotion regulation [10, 13]. Dissociation in BPD may therefore represent an active mechanism shaping symptom configuration, severity, and functional outcomes rather than merely an epiphenomenon of childhood trauma. This view is consistent with conceptualizations of dissociation as an adaptive attempt to distance oneself from overwhelming traumatic experiences and their associated memories [19].

AD, including depressive and anxiety disorders, is robustly associated with childhood trauma [20]. Longitudinal and population‐based studies show that early adverse experiences predict the development, persistence, and severity of internalizing psychopathology [7, 8], contributing to distress across diagnostic categories. Dissociative symptoms are also observed in AD and, although not a defining diagnostic feature, may complicate clinical presentation and course [4, 6, 21]. However, the strength of trauma‐dissociation associations across diagnostic groups remains unclear.

AD therefore provides an important contrast to BPD. Both disorders are associated with elevated rates of childhood trauma and trauma‐related psychopathology, yet they differ substantially in the prominence of dissociative symptoms [3, 18]. While dissociation represents a core and clinically relevant feature of BPD, it is typically less pronounced and not diagnostically central in AD [6]. Comparing BPD with AD allows examination of whether trauma–dissociation associations differ across clinically distinct treatment populations. Given the differing prominence of personality pathology in BPD and AD, this comparison may help clarify whether trauma–dissociation associations are more strongly expressed in the context of personality pathology or reflect processes that extend across diagnostic categories. Trauma‐dissociation links in BPD may therefore reflect features that are particularly salient in personality pathology while also being embedded within broader transdiagnostic mechanisms. Network psychometrics provide a framework for addressing this question. Rather than assuming latent disease entities, network models conceptualize psychopathology as interacting components [22], with dissociative symptoms functioning as dynamic elements within the system. Gaussian graphical models, estimated using partial correlations, allow examination of conditional associations between variables [23]. Dissociative symptoms may play an active role within broader psychopathological networks by interfering with cognitive and affective processing [10, 24]. Comparing networks across diagnostic groups can thus clarify whether trauma‐dissociation links differ between BPD and AD and inform debates on disorder‐specific versus transdiagnostic mechanisms.

### 1.1 Aims of the Study

The present study examined associations between childhood trauma and dissociation in individuals with BPD and AD. First, network models focusing on childhood trauma and dissociative symptoms were estimated and compared between groups. Second, trauma‐ and stress‐related symptoms as well as overall psychological distress were incorporated to examine whether the observed network structure remained stable within a broader symptom context. We hypothesized that individuals with BPD would report higher levels of childhood trauma and dissociation than individuals with AD. Given the limited evidence regarding diagnosis‐specific versus transdiagnostic mechanisms, group differences in network structure were examined on an exploratory basis.

## 2 Methods

### 2.1 Participants and Data Collection

Participants were recruited from clinical services in Greater London as part of two ongoing research projects initiated in 2013. Inclusion criteria were: age between 18 and 80 years and fluency in English. Exclusion criteria comprised: a diagnosis of schizophrenia or a recent psychotic episode, learning disability, and neurological disorder.

A total of 1389 individuals were screened. Of these, 569 were referred from specialist personality disorder services, including individuals with suspected or confirmed diagnoses of BPD or antisocial personality disorder (ASPD). Diagnoses were established using the Structured Clinical Interview for DSM‐IV Axis II Personality Disorders (SCID‐II; [25]) and the Personality Assessment Inventory‐ Borderline Features Scale (PAI‐BOR; [26]). Referral to these services required that personality pathology constituted the primary focus of assessment and treatment. The remaining 820 participants were recruited via National Health Service (NHS) Improving Access to Psychological Therapies (IAPT)/NHS Talking Therapies (TTad) services and were eligible if they reported clinically significant anxiety or depressive symptoms and qualified for low‐ or high‐intensity face‐to‐face therapy within IAPT/TTad. In line with the remit of these services, anxiety and/or depressive symptoms constituted the primary focus of treatment, although comorbid conditions were not systematically assessed or excluded. Both studies used a cross‐sectional design. PD participants attended two in‐person sessions at University College London, completing behavioral tasks, self‐report questionnaires, and structured interviews. IAPT/TTad participants completed a remote version of the study, which included the same self‐report measures and one behavioral task. All participants provided written informed consent and were compensated £10 per hour.

### 2.2 Measures

*The Brief Symptom Inventory* (BSI‐53; [27]) is a 53‐item self‐report instrument assessing general psychological distress during the past 7 days. Items are rated on a five‐point Likert scale ranging from “not at all” (0) to “extremely” (4). Although the BSI‐53 comprises nine primary symptom dimensions, the present study used only the Global Severity Index (GSI) as an overall measure of psychological distress. The GSI is calculated as the mean of all 53 items, with higher scores indicating greater overall symptom burden. The BSI‐53 has demonstrated good psychometric properties, including high internal consistency and satisfactory validity [27]. In the present study, the GSI exhibited good to excellent internal consistency (McDonald's *ω* = 0.95).

*The Childhood Trauma Questionnaire* (CTQ) is a 25‐item self‐report instrument comprising five subscales: emotional abuse, physical abuse, sexual abuse, emotional neglect, and physical neglect [28]. Each subscale consists of five items rated on a five‐point Likert scale ranging from “never true” (1) to “very often true” (5). The CTQ has demonstrated adequate psychometric properties [28, 29]. In the present study, internal consistency was good to excellent across subscales (McDonald's *ω* = 0.89 for emotional abuse, 0.90 for physical abuse, 0.95 for sexual abuse, 0.91 for emotional neglect, and 0.77 for physical neglect).

*The Dissociative Experiences Scale* (DES) [30, 31] is a 28‐item self‐report instrument assessing the frequency of dissociative experiences [30, 31]. Items are rated using percentage increments ranging from 0% to 100%, corresponding to an 11‐point Likert‐type scale. In line with the three‐factor structure proposed by Carlson et al. [32], subscales reflecting dissociative amnesia, derealisation/depersonalization, and absorption were used. The DES has demonstrated adequate psychometric properties in previous research [33, 34]. In the present study, internal consistency was good to excellent for the total scale and across subscales (McDonald's *ω* = 0.87 for amnesia, 0.87 for absorption, and 0.87 for derealization/depersonalization).

*The PTSD Checklist—Stressor‐Specific Version* (PCL‐S; [35]) is a 17‐item self‐report instrument assessing PTSD symptoms in accordance with DSM‐IV criteria. The items correspond to the three symptom clusters of re‐experiencing, avoidance/numbing, and hyperarousal. Respondents rate the extent to which they have been bothered by each symptom over the past month on a five‐point Likert scale ranging from “not at all” (1) to “extremely” (5). The PCL‐S has demonstrated good to excellent psychometric properties, including high internal consistency, satisfactory test–retest reliability, and strong convergent validity with clinician‐administered PTSD measures [36]. In the present study, internal consistency was good to excellent for the total scale and across subscales (McDonald's *ω* = 0.89 for re‐experiencing, 0.83 for avoidance/numbing, and 0.82 for hyperarousal).

### 2.3 Data Analysis

All statistical analyses were conducted using R version 4.2.3 [37]. For transparency and reproducibility, the R script is available online (https://osf.io/w8hm4/). Participants with BPD and AD were matched on age, gender, and overall symptom severity as indexed by the Global Severity Index (BSI‐GSI) using nearest‐neighbor propensity score matching with a 1:1 ratio and a caliper of 0.2, as implemented in the MatchIt package in R. Balance between groups was evaluated using standardized mean differences (SMDs), with values below 0.10 indicating adequate covariate balance [38]. The resulting matched sample was used for all subsequent analyses. Group differences for all primary outcomes were examined using Welch's *t*‐tests. Effect sizes were quantified using Cohen's d. To control for multiple testing across CTQ and DES outcomes, *p*‐values were adjusted using the Benjamini–Hochberg false discovery rate (FDR) correction.

Network analyses were conducted separately for the BPD and AD groups. In a primary model, regularized partial correlation networks were estimated to examine multivariate associations between childhood trauma (CTQ: Emotional Abuse, Physical Abuse, Sexual Abuse, Emotional Neglect, Physical Neglect) and dissociative symptoms (DES: Amnesia, Derealisation/Depersonalization, Absorption). In a second step, posttraumatic stress symptoms (PCL‐S: Re‐experiencing, Avoidance, Arousal) and general psychological distress (BSI‐GSI) were added to the network to evaluate the robustness of trauma–dissociation associations when embedded within a broader symptom context.

Networks were estimated as regularized partial correlation networks implemented as Gaussian graphical models (GGMs). Model estimation was performed using the graphical least absolute shrinkage and selection operator with model selection based on the Extended Bayesian Information Criterion and a tuning parameter of *γ* = 0.5 [23]. Networks were estimated and visualized using bootnet and qgraph. Community structure was identified using the Walktrap algorithm on a pooled reference network and applied to group‐specific networks. Bridge expected influence (BEI) was computed to assess inter‐community connectivity.

Node predictability was calculated as explained variance based on neighboring nodes [39]. Group differences in network structure were tested using the Network Comparison Test (NCT; [40]). Accuracy was assessed via nonparametric bootstrapping, and stability via case‐dropping procedures, with correlation stability coefficients quantifying robustness [23].

## 3 Results

### 3.1 Matching

In the matched sample (*N* = 429 per group), covariate balance between the BPD and AD groups was excellent. SMDs for age (SMD = −0.05), gender (all |SMDs| ≤ 0.04), and overall symptom severity as indexed by the BSI‐GSI (SMD = 0.07) were all well below the predefined threshold of 0.10, indicating adequate balance across matching variables.

### 3.2 Sample Characteristics

Participants with BPD had a mean age of 30.96 years (SD = 9.68), and those with ad 31.39 years (SD = 9.99). In the BPD group, 19.6% identified as male, 78.3% as female, and 2.1% as other; in the AD group, 21.0%, 77.2%, and 1.9%, respectively. Mean BSI‐GSI was 1.94 (SD = 0.74) in BPD and 1.88 (SD = 0.68) in AD.

### 3.3 Group Differences in Trauma, Dissociation, and PTSD Symptoms

Welch's *t*‐tests compared CTQ, DES, and PCL‐S scores between groups. Individuals with BPD reported higher childhood trauma across all CTQ subscales and total score (all FDR‐adjusted *p* < 0.001; |d| = 0.43–0.73; Table 1). Dissociative symptoms were likewise higher across all DES subscales and total score (all FDR‐adjusted *p* < 0.001; |d| = 0.42–0.61). For PTSD symptoms, group differences were found for avoidance, arousal, and total score (FDR‐adjusted *p* ≤ 0.001; |d| = 0.26–0.30), whereas re‐experiencing was not significant (*p* = 0.096).

**TABLE 1:** Group comparisons on CTQ‐, DES‐, PCL‐S‐, and BSI Scales between patients with BPD (*N* = 429) and ADD (*N* = 429).

Variable	BPD	AD	*t*	*p* (FDR)	d
	M (SD)	M (SD)			
CTQ—Emotional abuse	16.75 (5.79)	12.82 (5.90)	−9.85	< 0.001	−0.67
CTQ—Physical abuse	10.17 (5.93)	7.92 (4.33)	−6.33	< 0.001	−0.43
CTQ—Sexual abuse	10.28 (6.84)	7.66 (5.33)	−6.25	< 0.001	−0.43
CTQ—Emotional neglect	16.62 (5.35)	12.98 (5.44)	−9.89	< 0.001	−0.68
CTQ—Physical neglect	10.48 (4.53)	8.38 (3.76)	−7.37	< 0.001	−0.50
CTQ—Total score	64.42 (21.67)	49.77 (18.10)	−10.71	< 0.001	−0.73
DES—Amnesia	20.87 (20.07)	13.17 (16.23)	−6.18	< 0.001	−0.42
DES—Derealisation/depersonalization	30.36 (25.02)	16.87 (19.54)	−8.80	< 0.001	−0.60
DES—Absorption	47.05 (22.41)	36.39 (21.91)	−7.05	< 0.001	−0.48
DES—Total score	35.01 (18.82)	24.22 (16.62)	−8.86	< 0.001	−0.61
PCLS—Re‐experiencing	17.47 (5.30)	16.81 (5.69)	−1.76	0.096	−0.12
PCLS—Avoidance	23.93 (6.76)	21.83 (7.15)	−4.43	< 0.001	−0.30
PCLS—Arousal	17.98 (4.87)	16.63 (5.46)	−3.84	0.001	−0.26
PCLS—Total score	59.39 (14.37)	55.27 (16.55)	−3.89	0.001	−0.27

*Note: p*‐values are adjusted using the Benjamini–Hochberg false discovery rate (FDR) correction. Effect sizes represent Cohen's d.

### 3.4 Network‐Analyses

The following network analyses are based on regularized partial correlation networks, in which edges represent conditional associations between variables after controlling for all other nodes in the network. Node importance was evaluated using BEI, reflecting connectivity within and between symptom communities, respectively.

The core trauma‐dissociation networks were accurate and stable in both groups. Case‐dropping bootstrap analyses indicated high edge stability (BPD: CS = 0.75; AD: CS = 0.75). Nonparametric bootstrapping indicated satisfactory edge accuracy for both groups (Figures S1 and S2). Mean node predictability was moderate (BPD: 51%; AD: 45%), suggesting that neighboring nodes accounted for approximately half of the variance in individual network nodes. The Walktrap algorithm identified two communities (CTQ, DES). The BPD network showed 19 nonzero edges (mean = 0.12), the AD network 15 (mean = 0.11; Figure 1), indicating slightly higher density in BPD. Associations were almost exclusively positive in both networks; the only negative edge was a weak association between DES Amnesia and CTQ Emotional Neglect (edge weight = −0.02) in the BPD network. Inter‐community connectivity between the CTQ and DES communities (BEI), is shown in Figure 2.

**FIGURE 1:**
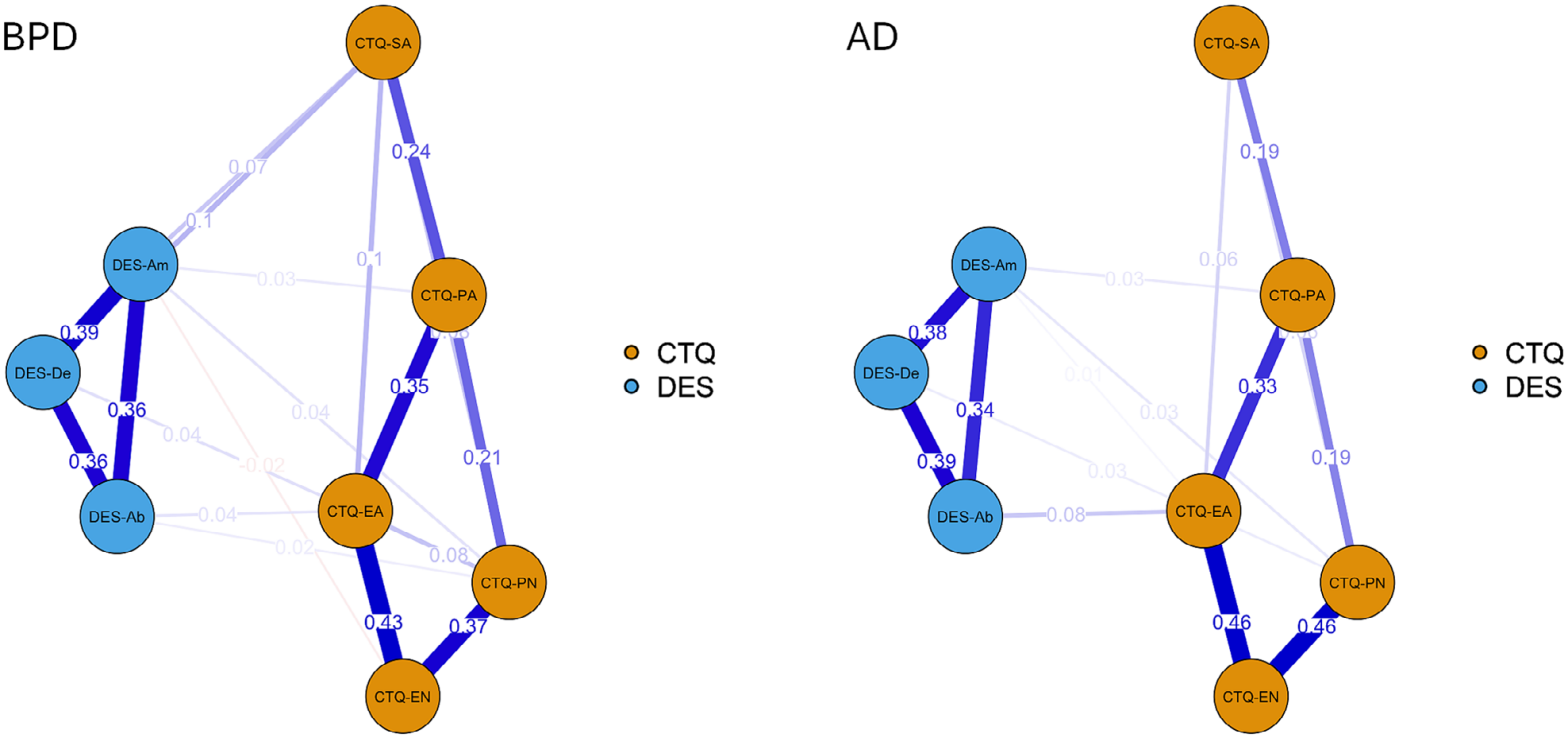
Core networks for the BPD (left) and AD (right) groups. Edge thickness reflects the strength of the association, and color indicates direction, with blue representing positive and red representing negative associations. Node colors indicate community membership as determined by the Walktrap algorithm.

**FIGURE 2:**
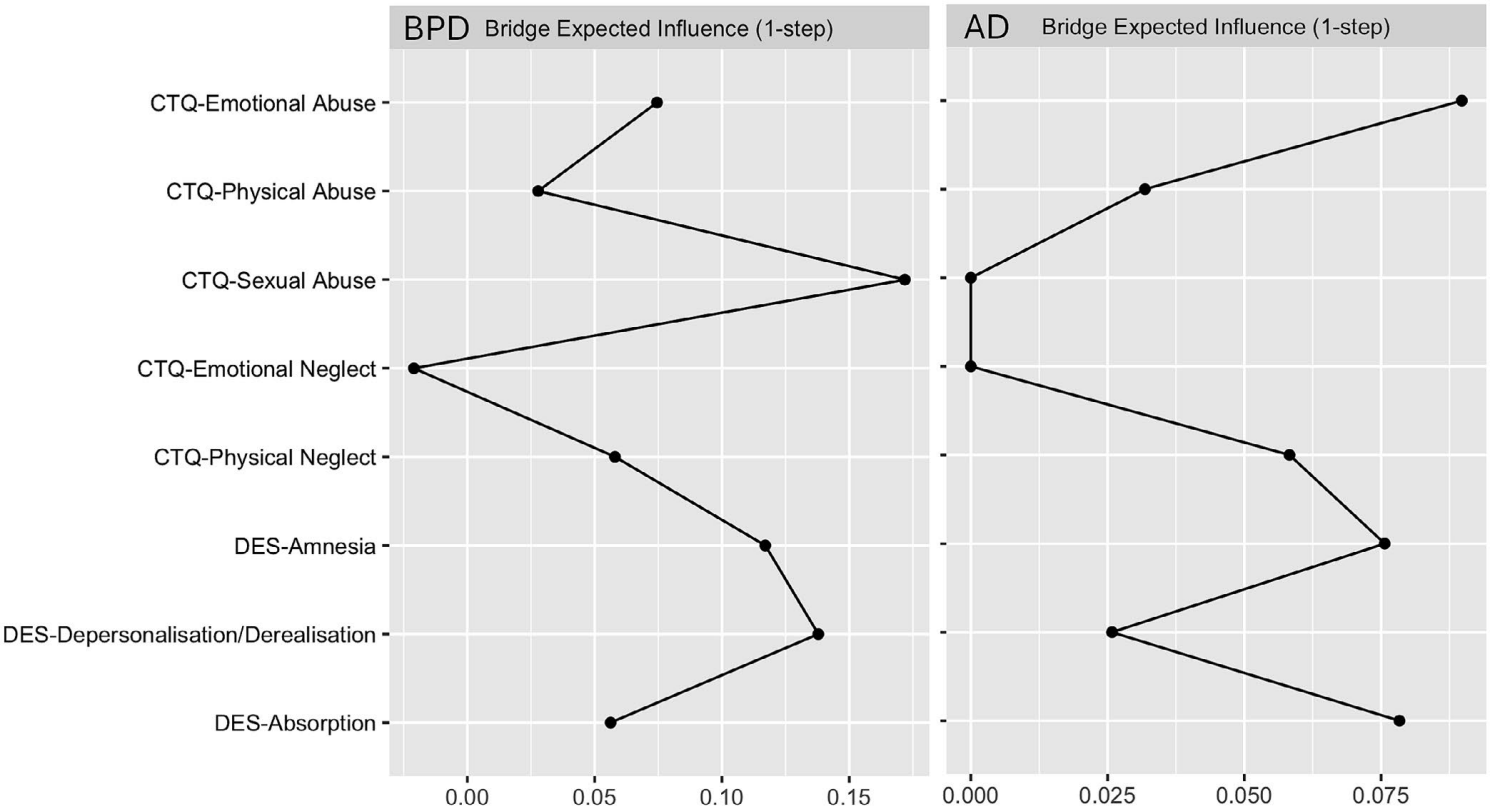
BEI for the core CTQ–DES networks in the BPD (left panel) and AD (right panel) groups.

The NCT indicated no group differences (M = 0.10, *p* = 0.739). Global strength was also nonsignificant (S = 0.29, *p* = 0.091). Thus, no evidence was found for global structural differences between groups in the core trauma–dissociation network. Given the non‐significant global tests and the hierarchical nature of the NCT procedure, no evidence was found for meaningful differences in network structure or overall connectivity between groups. At the node level, only Sexual Abuse showed a significant difference in bridge expected influence (*p* = 0.032, FDR‐corrected).

The extended networks including PTSD symptoms and global distress were also stable. Edge stability was high in both groups (BPD: CS = 0.75; AD: CS = 0.75; Figures S3 and S4), and centrality stability acceptable to high (BPD: CS = 0.52; AD: CS = 0.75). Predictability remained moderate (BPD: 51%; AD: 51%). Three communities emerged: CTQ, DES, and a combined PCL‐S/BSI‐GSI cluster. The BPD network showed 36 nonzero edges, the AD network 37 (both mean = 0.08; Figure 3). Associations were almost exclusively positive. The only negative edge was a negligible association between DES Amnesia and CTQ Emotional Neglect in the BPD network (edge weight = −0.01). Network comparison revealed no differences in structure (M = 0.14, *p* = 0.304) or global strength (S = 0.19, *p* = 0.299). The previously observed difference in BEI for Sexual Abuse was no longer significant (*p* = 0.059; Figure S5).

**FIGURE 3:**
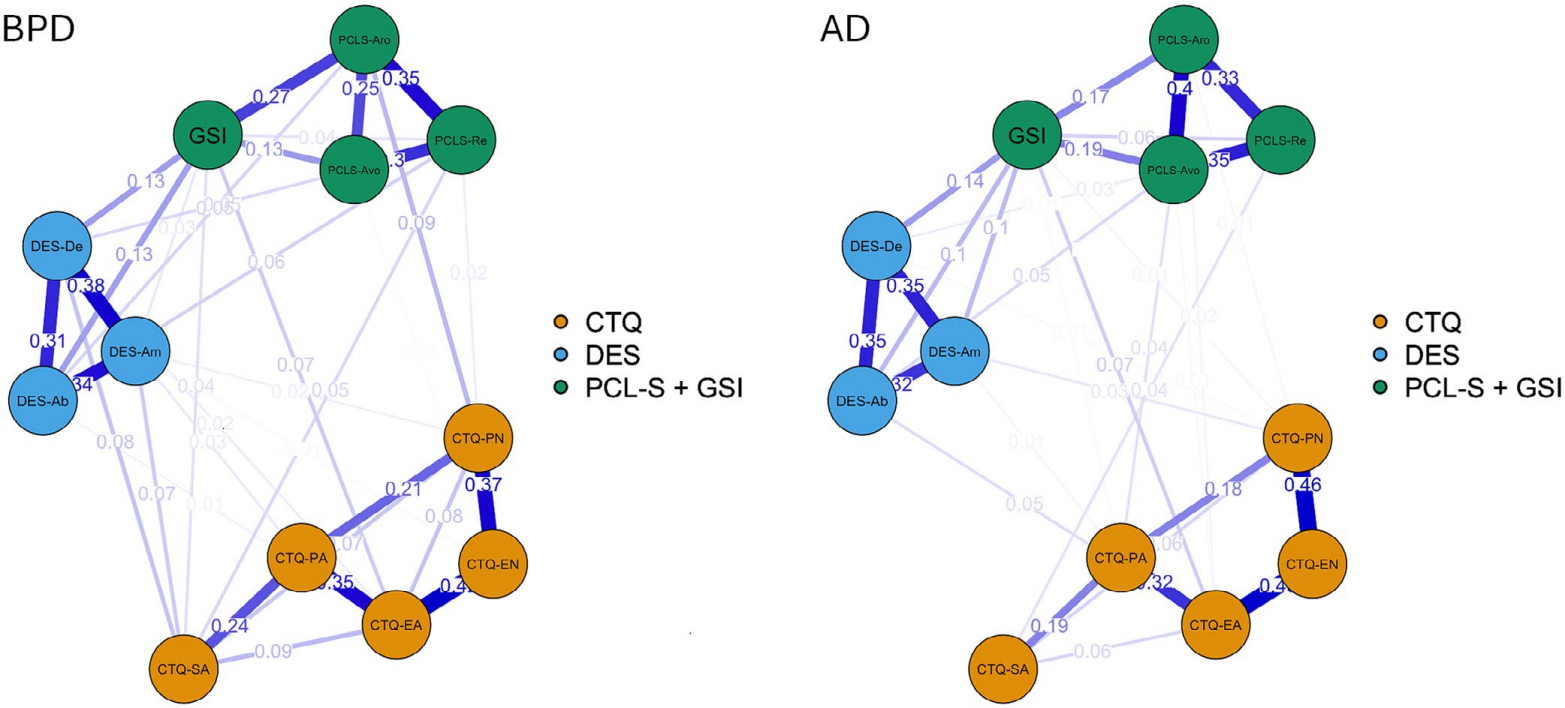
Extended networks for the BPD (left) and AD (right) groups.

## 4 Discussion

The present study examined whether associations between childhood trauma and dissociation differ structurally between BPD and AD, using regularized partial correlation networks to model conditional dependencies while controlling for overall symptom severity. As expected, individuals with BPD reported higher levels of childhood trauma (across all CTQ subscales) and dissociative symptoms than those with AD, even after propensity score matching on age, gender, and overall symptom severity—a finding that aligns with meta‐analytic evidence of elevated trauma exposure (OR = 14) and dissociative prevalence in BPD [9, 10, 11, 12, 17]. Critically, however, NCT revealed no significant differences in global network structure, edge weights, or centrality indices between groups, neither in the core trauma–dissociation network nor in the extended model incorporating PTSD symptom clusters and distress.

These findings suggest that BPD reflects greater severity within a shared trauma‐related network rather than a distinct, disorder‐specific architecture. Importantly, this pattern was not limited to the core trauma–dissociation network. The same findings emerged in the extended network including PTSD symptom clusters and global psychological distress, where network structure and overall connectivity remained comparable across groups. This suggests that the observed architecture is not specific to childhood trauma and dissociation alone, but remains largely preserved when embedded within a broader symptom context. This distinction is conceptually important, as it challenges etiological models positing unique trauma–dissociation pathways in BPD. Contemporary models conceptualize BPD within a diathesis‐stress framework, emphasizing heightened emotional and stress sensitivity as well as stress‐related dissociation [12, 41]. If these processes were organized in a disorder‐specific manner, one would expect differences in how trauma and dissociation are interrelated in BPD compared to AD. However, the present findings instead support a severity‐within‐architecture framework, in which BPD reflects amplified expression within a common vulnerability system, with trauma exposure predicting dissociative symptoms transdiagnostically. Consistent with recent transdiagnostic network studies (e.g., [17, 42, 43]), these findings suggest that dissociation occupies a central position within the network and may help explain how childhood trauma is associated with a broader range of trauma‐related symptoms in both disorders.

Null findings should be interpreted cautiously. The NCT is an omnibus procedure with limited sensitivity to small or localized effects, particularly in regularized models [40]. Accordingly, nonsignificant results do not imply structural equivalence, but rather that no differences exceeding the detectable threshold were observed. Against this backdrop, a localized effect emerged: sexual abuse showed elevated bridge expected influence in BPD within the core network, even after FDR correction. This pattern may indicate a subtype‐specific variation that becomes less detectable in higher‐dimensional networks. While preliminary, it is consistent with prior work suggesting that different forms of childhood trauma may occupy distinct positions within psychopathological networks [44]. However, given that the effect did not replicate in the extended network, any interpretation of its clinical or theoretical significance remains tentative.

The present findings have implications for both etiological theory and clinical practice. If trauma, dissociation, and PTSD‐related domains are organized within a largely shared conditional structure across BPD and AD, this challenges overly diagnosis‐specific causal models that position trauma‐related processes as uniquely configured in BPD. Instead, BPD may reflect elevated loadings within a common trauma‐related vulnerability system. Clinically, this perspective lends support to transdiagnostic, mechanism‐focused interventions targeting shared processes such as trauma processing/mentalizing, dissociation management, and affect regulation (e.g., EMDR, dissociation‐focused skills in DBT, trauma‐focused MBT, or phase‐based treatments) [45, 46, 47]. While individuals with BPD may present with greater severity and complexity, this may be addressed through intensified treatment dosing or comorbidity‐informed protocols rather than fundamentally distinct mechanistic models.

Several limitations should be acknowledged. First, the cross‐sectional design precludes causal inference. Network edges reflect conditional associations rather than directional effects, and longitudinal or intensive time‐series data would be required to examine dynamic processes. Second, trauma exposure was assessed retrospectively using self‐report, which may be subject to recall bias [48].

Third, although propensity score matching enhanced comparability across groups, the AD sample did not undergo structured diagnostic interviews and PDs were not formally excluded. Likewise, affective disorders were not systematically assessed in the BPD sample. Participants were recruited from routine clinical services according to the primary focus of assessment and treatment rather than as diagnostically pure groups. Admission to each treatment pathway required that the respective clinical presentation constituted the primary focus of care, although comorbid conditions were not systematically assessed or excluded. Diagnoses were established as part of routine clinical practice by qualified mental health professionals, but formal inter‐rater reliability was not assessed. As such, the present findings should be interpreted within the context of naturalistic clinical samples, in which diagnostic heterogeneity and psychiatric comorbidity are common and may have contributed to the observed similarities in network structure. Fourth, regularized Gaussian graphical models prioritize sparse solutions and may obscure weak, but potentially meaningful edges. Finally, although the matched sample was substantial, the NCT may not detect subtle local differences; thus, small architecture variations cannot be ruled out. Future studies may benefit from incorporating the broader DSM‐5 PTSD symptom structure as well as disturbances in self‐organization associated with complex PTSD. Such approaches may help clarify the extent to which trauma‐related symptom networks differ across BPD, PTSD, and complex PTSD, and further inform ongoing discussions regarding their overlap and distinctiveness (e.g., [49]).

In summary, individuals with BPD reported substantially higher levels of childhood trauma and dissociative symptoms than individuals with AD. However, the conditional dependency structure linking trauma, dissociation, PTSD symptoms, and global distress was largely comparable across diagnoses. These findings support a severity‐within‐architecture account: BPD appears characterized by elevated magnitude within a broadly shared trauma‐related network configuration, rather than by qualitatively distinct and BPD‐unique interrelations. By distinguishing between differences in symptom level and differences in structural configuration, the present study contributes to ongoing debates regarding diagnosis‐specific versus transdiagnostic mechanisms. The results suggest that trauma‐related processes may operate within a common architecture across diagnostic categories, while leaving open the possibility that additional, disorder‐specific processes operate outside the domains modeled here.

## Author Contributions

P.W., C.S., and T.N. conceptualized the study and P.W. performed data analyses. P.W. drafted the first manuscript. T.N. coordinated data acquisition and curation. P.R.M. and P.F. secured funding. J.L. and S.P. oversaw data collection. All authors reviewed the manuscript critically and contributed to the manuscript.

## Funding

Philipp Wülfing received funding from the Landesgraduiertenförderung Mecklenburg‐Vorpommern. C.S. received funding from the German Federal Ministry for Economic Affairs and Climate Action (Bundeshaushalt 2021, Kapitel 0910, Titel 68603, and Bundeshaushalt 2024/25, Kapitel 0415, Titel 544 01 Förderzeichen 411‐AS 06/2024). The funders had no role in study design, data collection, data analysis, data interpretation or writing of this article. The authors certify that there are no financial interests to declare. This work was supported by a National Institute for Health Research (NIHR) Senior Investigator Award (NF‐SI‐0514‐10157) awarded to Peter Fonagy. The authors also acknowledge funding by NIH‐NIDS (5R01NS092701‐03) and a Wellcome Trust Principal Research Fellowship awarded to Read Montague.

## Disclosure

The authors have nothing to report.

## Conflicts of Interest

The authors declare no conflicts of interest.

## Supporting information


**Figure S1:** Bootstrapped confidence intervals for edge weights in the DES‐CTQ BPD network.

**Figure S2:** Bootstrapped confidence intervals for edge weights in the DES‐CTQ AD network.

**Figure S3:** Bootstrapped confidence intervals for edge weights in the extended BPD network.

**Figure S4:** Bootstrapped confidence intervals for edge weights in the extended AD network.

**Figure S5:** BEI for the extended CTQ–DES‐PCLS‐GSI networks in the BPD (left panel) and AD (right panel) groups.

## Data Availability

The Supporting Informations and R code have been made publicly available at the Open Science Framework and can be accessed at https://osf.io/w8hm4/. For ethical reasons, the data from this study are available only upon reasonable request from the last author.
